# Supplementary material for: Modulation of the 5-Lipoxygenase Pathway by Chalcogen-Containing Inhibitors of Leukotriene A4 Hydrolase
Source: Int J Mol Sci. 2023 Apr 19;24(8):7539. doi: 10.3390/ijms24087539 (PMC10145651; doi:10.3390/ijms24087539)
Supplement: Supplementary file 1 [file ijms-24-07539-s001.zip › ijms-2327425-supplementary.pdf]

SUPPLEMENTARY INFORMATION

"Modulation of the 5-lipoxgenase pathway by chalcogen-containing inhibitors of leukotriene A4 hydrolase."

Tarvi Teder, Stefanie König, Rajkumar Singh, Bengt Samuelsson,
Oliver Werz, Ulrike Garscha and Jesper Z. Haeggström

Figure S1. LC-MS and NMR spectra of TTSe. TTSe was synthesized by LifeChemicals Co. and the compound was identified by LC-MS (A) and NMR approach (B).

Figure S2. LC-MS and NMR spectra of TTO. TTO was synthesized by LifeChemicals Co. and the compound was identified by LC-MS (A) and NMR approach (B).

Figure S3. **Cytotoxicity assays with chalcogen-containing inhibitors and leukocytes.** MTT and LDH assays were carried out with peripheral blood-derived monocytes and polymorphonuclear neutrophils (PMNs), respectively. The cell viability of monocytes after 24 hours and the release of LDH from membranes of PMNs after 30 minutes were altered only with at high concentrations of inhibitors. * p-value < 0.05; *** p-value < 0.001; ns - non-significant difference.

Table S1. Predicted pharmacological properties of chalcogen-containing inhibitors. Individual atomic properties of chalcogens, and bioavailability and toxicity of the inhibitors were determined.

Figure S4. Inhibition assay with soluble epoxide hydrolase and chalcogen-containing inhibitors. Although ARM1 and TTSe did not have any effect on the activity of soluble epoxide hydrolase (sEH), 10 μM of TTO attenuated the activity of sEH. * p-value < 0.05; non-significant differences are not indicated.

Figure S5. Levels of LTB4 and 5S-HETE produced in the incubations with exogenous arachidonic acid in the presence of chalcogen containing inhibitors. The levels of LTB4 and 5S-HETE were determined from polymorphonuclear neutrophils (PMNs).

Table S2. Peptidase activity with different peptide derivatives.

Figure S8. Crystal and diffraction map of the LTA_4H-TTSe complex.

Figure S9. Crystal and diffraction map of the LTA_4H-TTSe complex.

Table S3. Data collection, refinement, and model building statistics of LTA_4H in complex with TTSe and TTO.

Figure S8. Superimposition of selective inhibitors co-crystallized in a complex with LTA_4H. ARM1 (PDB ID: 4L2L), 4OMe-ARM1 (PDB ID: 6O5H) and 4MDM (7LLQ) are colored as blue, brown and pink, respectively.

Figure S9. Preparation of recombinant LTA_4H. LTA4H on Mono Q (A) and Superdex 16/600 (B) columns prior to the crystallization. SDS-PAGE image (C) showing the purity of 1 μg (1) and 10 μg (2) of LTA_4H.

Figure S10. Standard curves for free proline, para-nitroanilide and 7-amido-4-methylcoumarin. Standard curves of free proline (A), para-nitroanilide (pNa) (B) and 7-amido-4-methylcoumarin (AMC) (C) were used to calculate specific activities of LTA_4H with Pro-Gly-Pro and peptide analogues.

Fig. S1

A

Agilent 1100 LC/MSD SL
Diodearrey G1315B (DAD1A-215nm; DAD1B-241nm)
Mass Quad G1956B (MSD1-Pos, MSD2-Neg)
ELSD Altech 3300 (ADC1 A, ELSD)

Mobile Phase:A-H2O+0.1%HCOOH;B-MeCN+0.1%HCOOH
Separation colu: 100 %
Rapid Resolutionn HT Cartrige 4.6x30mm,
1.8-Micron, Zorbax SB C-18

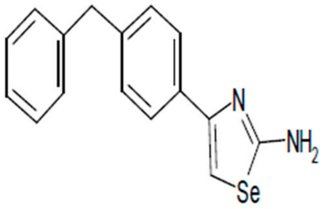

Mol.Weight: 313.26
Salt:

F0006-0446

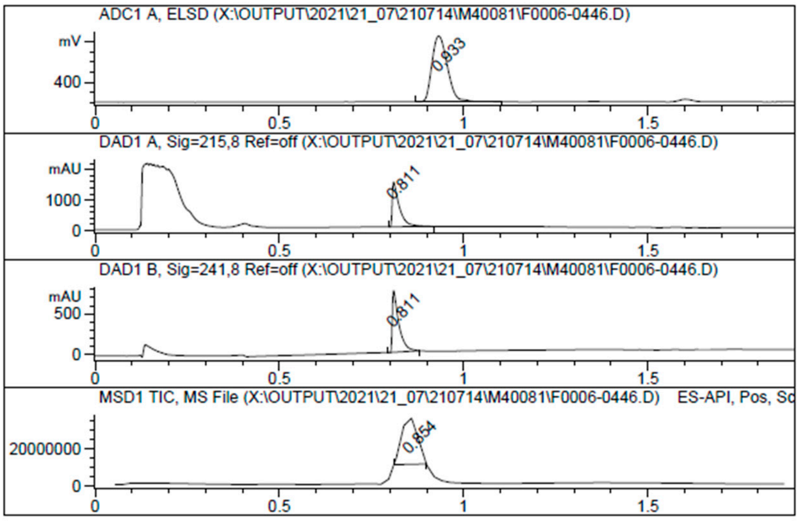

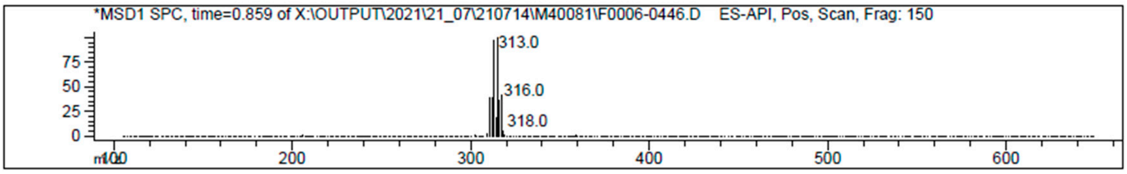

#	Signal	R.Time	Area %
1	ADC1 A, ELSD	0.933	100.000

#	Signal	R.Time	Area %
1	DAD1 A, Sig=215,8 Ref=off	0.811	100.000

#	Signal	R.Time	Area %
1	DAD1 B, Sig=241,8 Ref=off	0.811	100.000

#	Signal	R.Time	Area %
1	MSD1 TIC, MS File	0.854	100.000

B

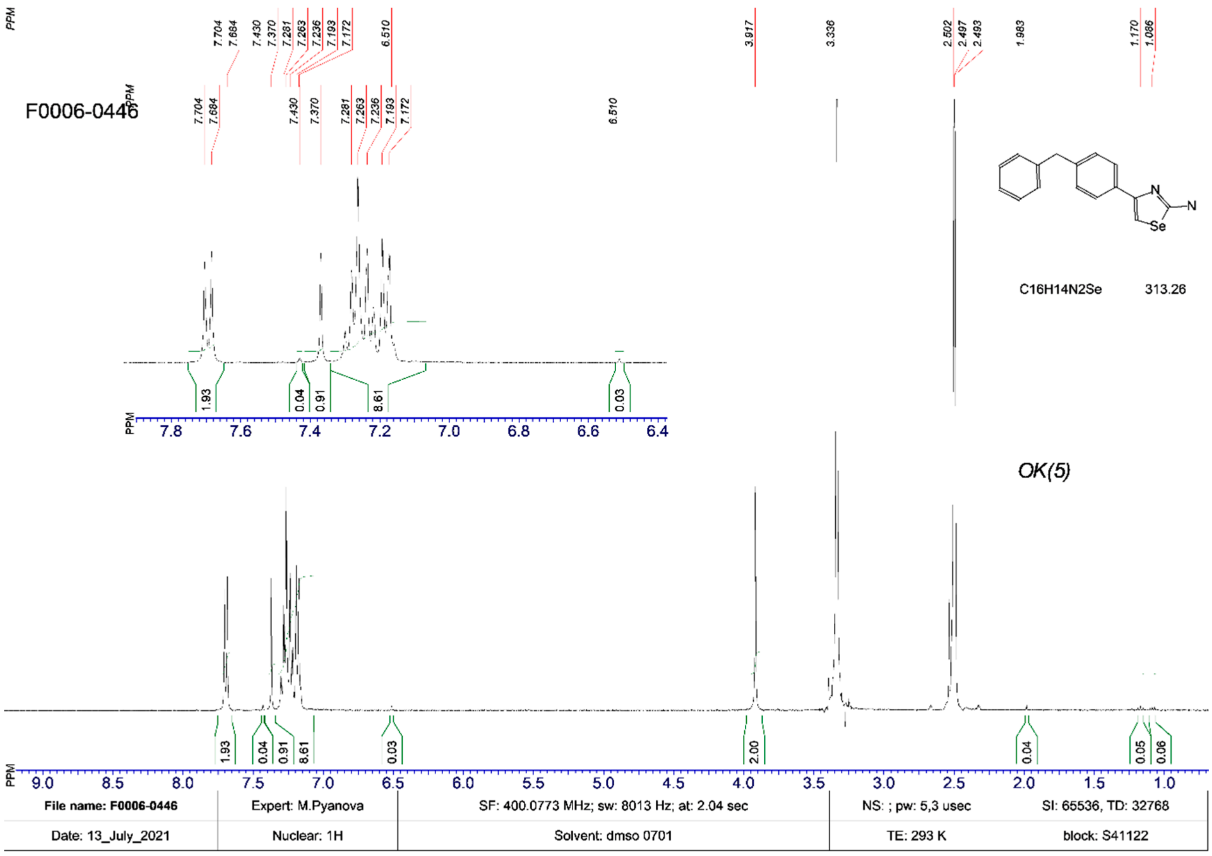
F0006-0446
C16H14N2Se 313.26
OK(5)
File name: F0006-0446
Expert: M.Pyanova
SF: 400.0773 MHz; sw: 8013 Hz; at: 2.04 sec
NS: ; pw: 5,3 usec
SI: 65536, TD: 32768
Date: 13_July_2021
Nuclear: 1H
Solvent: dmso 0701
TE: 293 K
block: S41122

Fig. S2.

A

```
Agilent 1100 LC/MSD SL                                 Mobile Phase:A-H2O+0.1%HCOOH;B-MeCN+0.1%HCOOH
Diodearrey G1315B (DAD1A-215nm; DAD1B-241nm)          Separation column:        100 %
Mass Quad  G1956B (MSD1-Pos,   MSD2-Neg)              Rapid Resolutionn HT Cartrige 4.6x30mm,
ELSD Altech 3300  (ADC1 A, ELSD)                      1.8-Micron, Zorbax SB C-18
```

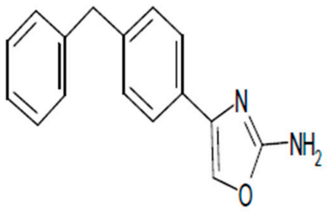

Mol.Weight: 250.3
Salt:

F0006-0445

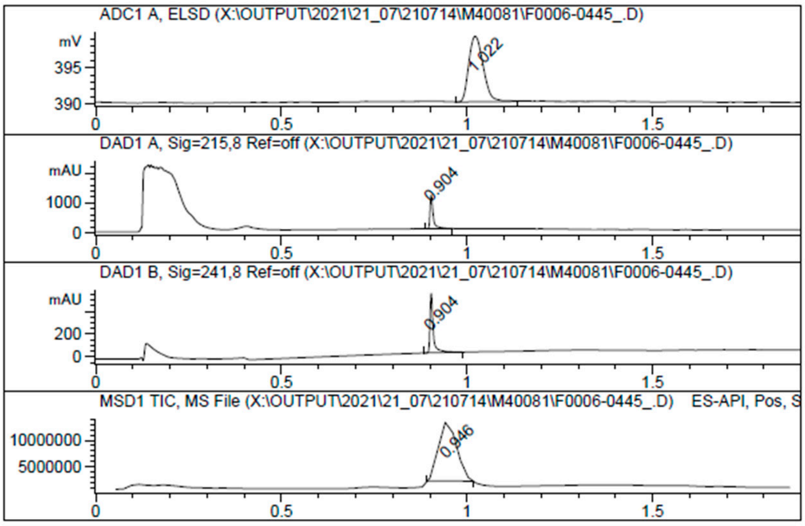

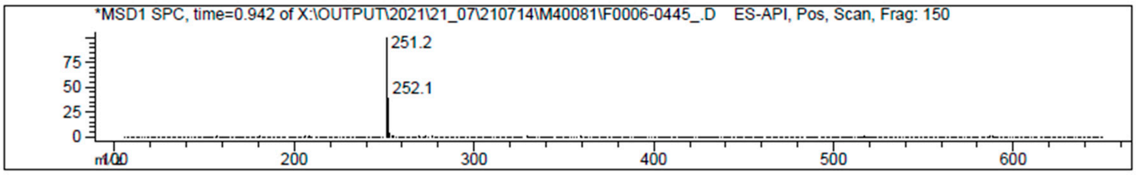

#	Signal	R.Time	Area %
1	ADC1 A, ELSD	1.022	100.000

#	Signal	R.Time	Area %
1	DAD1 A, Sig=215,8 Ref=off	0.904	100.000

#	Signal	R.Time	Area %
1	DAD1 B, Sig=241,8 Ref=off	0.904	100.000

#	Signal	R.Time	Area %
1	MSD1 TIC, MS File	0.946	100.000

B

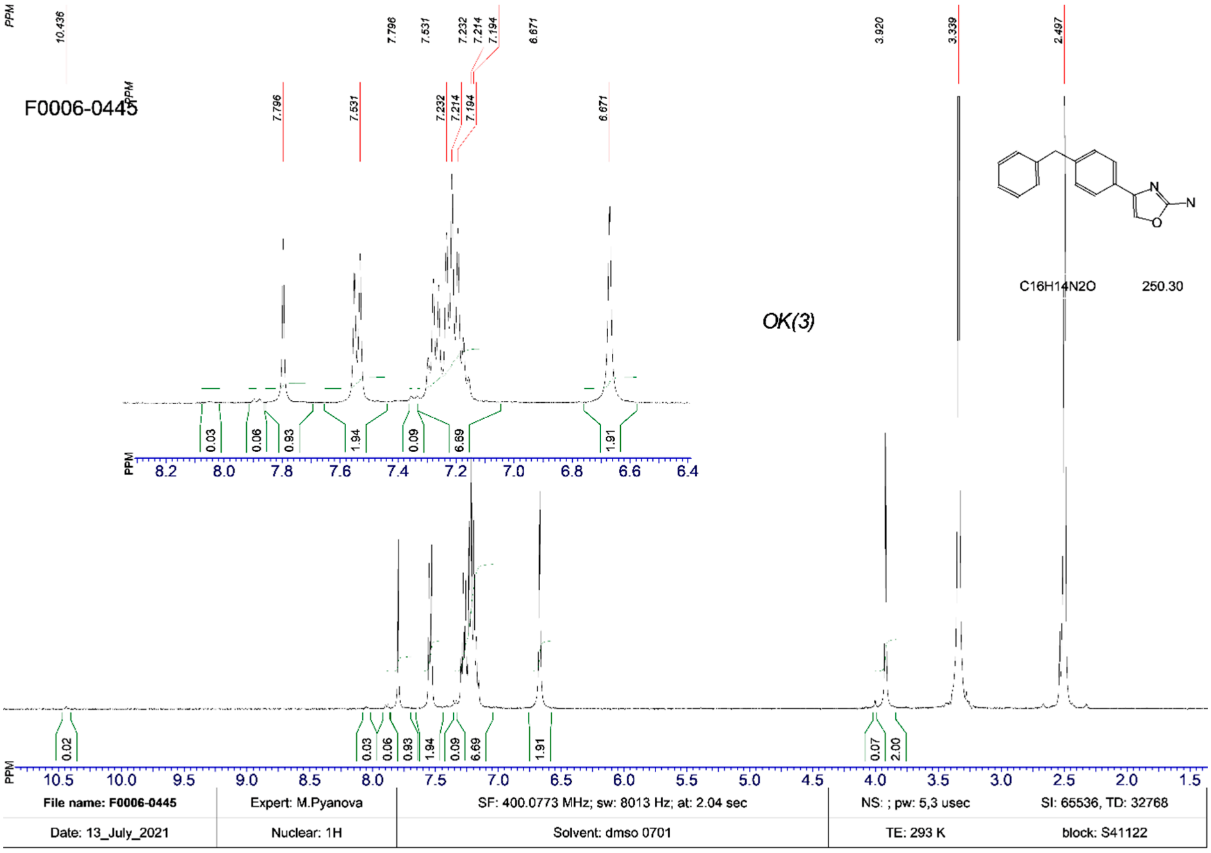
F0006-0445
OK(3)
C16H14N2O 250.30
File name: F0006-0445
Expert: M.Pyanova
SF: 400.0773 MHz; sw: 8013 Hz; at: 2.04 sec
NS: ; pw: 5,3 usec
SI: 65536, TD: 32768
Date: 13_July_2021
Nuclear: 1H
Solvent: dmso 0701
TE: 293 K
block: S41122

Fig. S3.

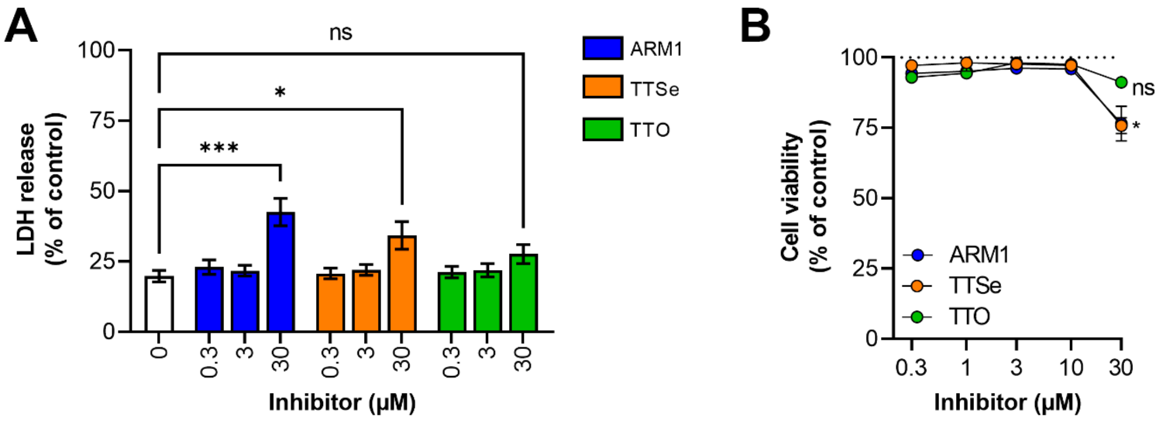
A
100
75
50
25
0
LDH release
(% of control)
ns
*
***
ARM1
TTSe
TTO
0
0.3
3
30
0.3
3
30
0.3
3
30
Inhibitor (μM)
B
100
75
50
25
0
Cell viability
(% of control)
ns
*
ARM1
TTSe
TTO
0.3
1
3
10
30
Inhibitor (μM)

Table S1.

ATOM		Oxygen	Sulfur	Selenium
Atomic properties	Atomic radius	60	100	115
	Van der Waals radius	155	180	190
	Covalent radius	66	105	120
	Electronegativity	3,44	2,58	2,55
**INHIBITORS**		**TTO**	**ARM1**	**TTSe**
Physical properties	MW	250	266	314
	cLogP	3.49	4.17	3.86
	Solubility	-4.9	-4.91	-3.25
	GI absorption	high	high	high
	BBB permeant	yes	yes	yes
	LogD pH 2.0	2.76	3.18	3.69
	LogD pH 3.0	3.4	3.63	3.87
	LogD pH 4.0	4.12	4.29	3.89
	LogD pH 7.0	4.45	4.61	3.9
	LogD pH 12.0	4.45	4.61	3.89
	TPSA	38.91	67.15	52.05
	Druglikeness	-3.24	2.87	0.3
	Drug score	0.40	0.61	0.53
Toxicity	Mutagenic	none	none	none
	Tumorigenic	none	none	none
	Irritant	none	none	none
	Reproductive effect	none	none	none

Radii are presented in picometers. Electronegativity is based on the Pauling scaling. MW – molecular weight; cLogP – the calculated partition coefficient of a compound; BBB – the blood-brain barrier; LogD – the distribution coefficient of a compound; TPSA – topological polar surface area.

Fig. S4.

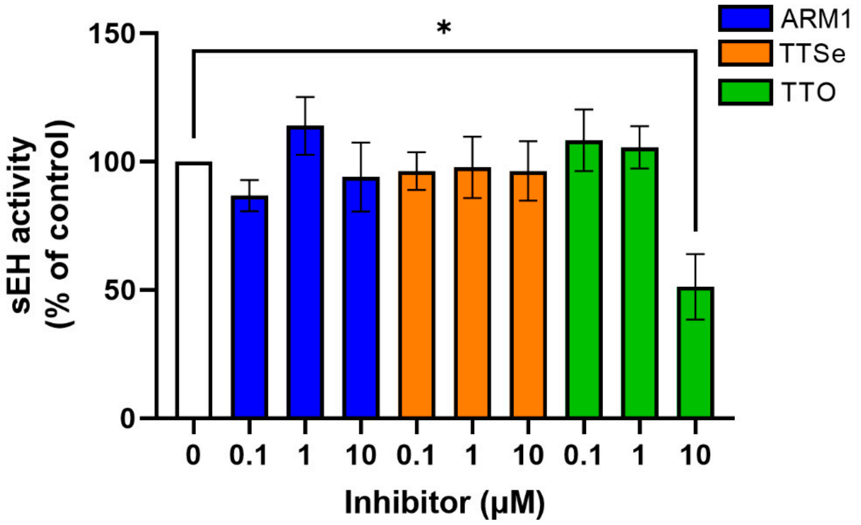
*
ARM1
TTSe
TTO
150
100
50
0
sEH activity
(% of control)
0
0.1
1
10
0.1
1
10
0.1
1
10
Inhibitor (μM)

Fig. S5.

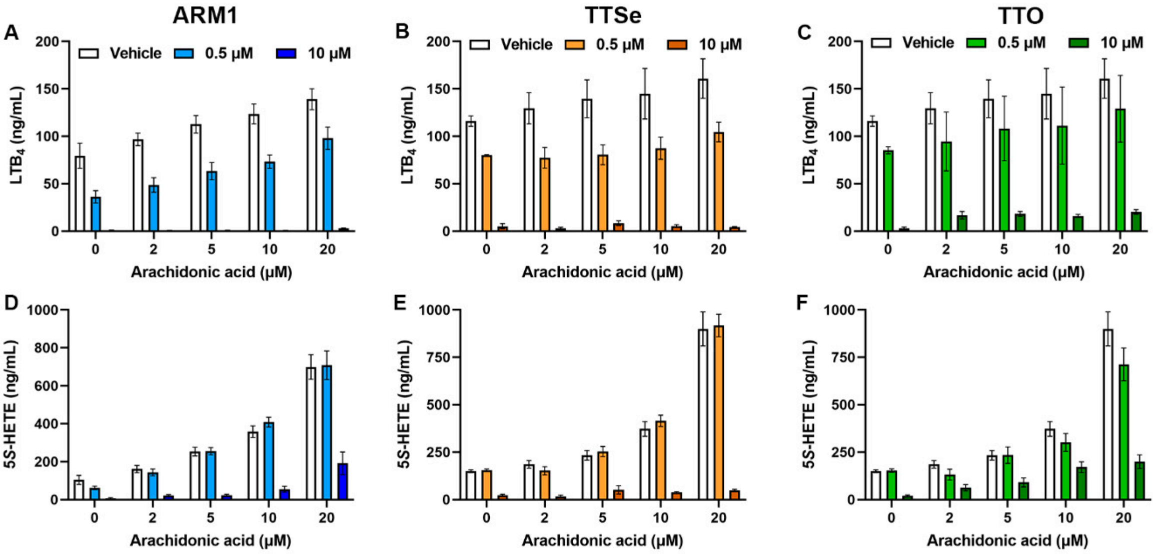

ARM1
TTSe
TTO
A
B
C
D
E
F
Vehicle
0.5 μM
10 μM
LTB4 (ng/mL)
5S-HETE (ng/mL)
Arachidonic acid (μM)

Table S2.

**Substrate**	**Specific activity (µmol/µg/min)**			
	**LTA_4H**	**ARM1**	**TTSe**	**TTO**
**L-Leu-pNa**	3.9 ± 0.1	46.3 ± 1.7 (12x)	37.6 ± 1.1 (10x)	46.9 ± 1.7 (12x)
**L-Ala-pNa**	10.3 ± 0.8	79.6 ± 7.6 (8x)	88.0 ± 4.6 (9x)	93.0 ± 1.7 (9x)
**L-Val-pNa**	0.8 ± 0.1	24.9 ± 0.8 (30x)	24.6 ± 0.5 (30x)	24.4 ± 0.4 (30x)
**L-Arg-pNa**	18.7 ± 1.2	0.20 ± 0.03 (94x)	0.20 ± 0.07 (94x)	0.2 ± 0.1 (89x)
**L-Lys-pNa**	2.5 ± 0.1	nd	nd	nd
**L-Pro-pNa**	3.0 ± 0.3	2.65 ± 0.12 (1x)	2.8 ± 0.1 (0.8x)	2.3 ± 0.2 (1.3x)
**L-Arg-AMC**	31.3 ± 2.2	10.1 ± 0.1 (3x)	10.1 ± 0.1 (3x)	9.9 ± 0.1 (3x)
**L-Pro-AMC**	31.3 ± 0.4	16.0 ± 0.2 (2x)	15.7 ± 0.2 (2x)	15.8 ± 0.2 (2x)

nd – not determined; data is presented as mean ± SEM with n = 3.

Fig. S6.

A

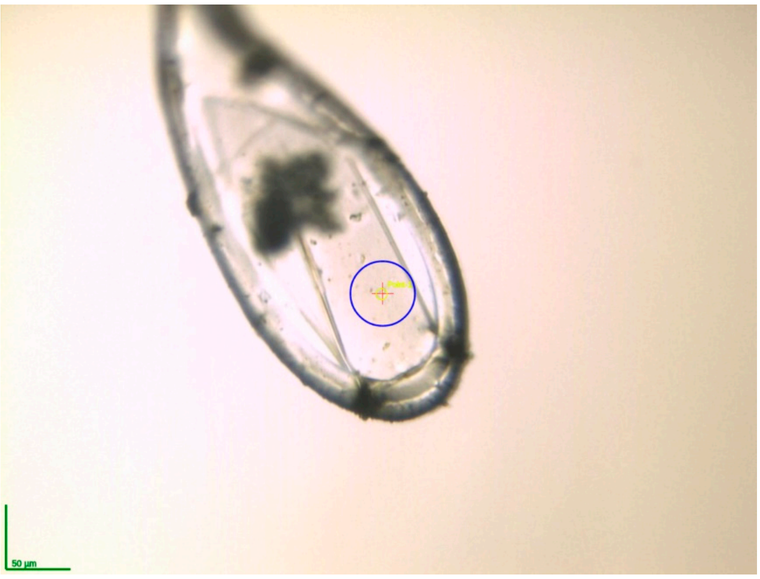

B

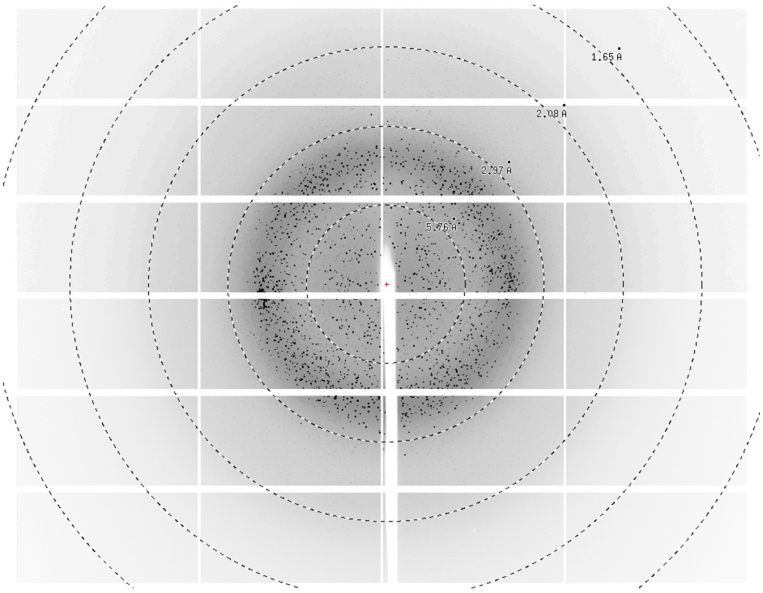

Fig. S7.

A

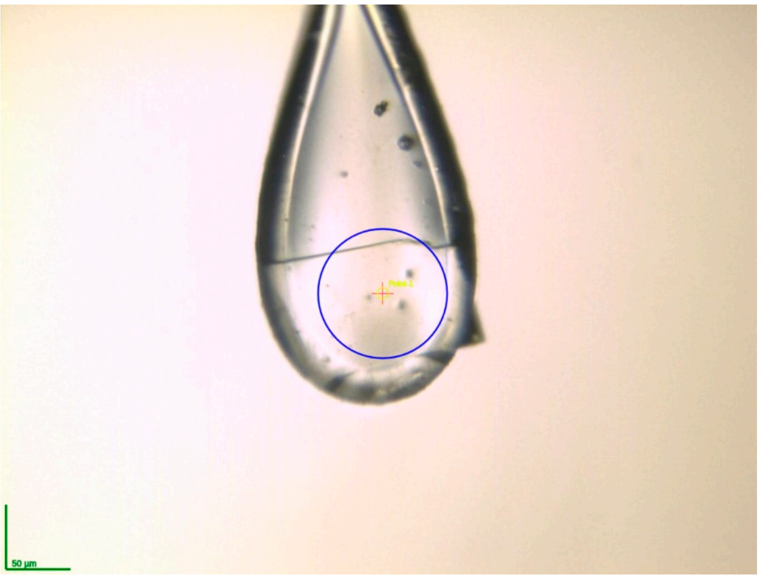

B

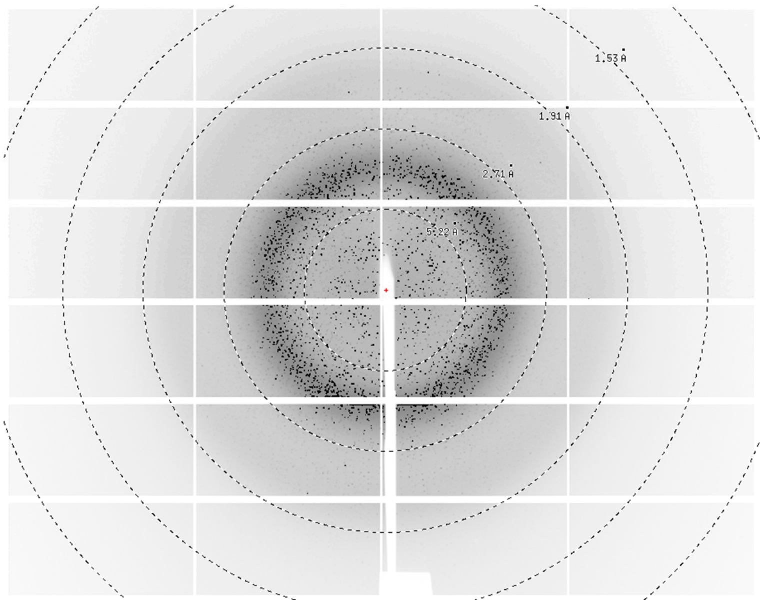

Table S3.

	**LTA_4H-TTSe**	**LTA_4H-TTO**
**Data collection**		
Space group	P 21 21 21	P 21 21 21
Cell dimension		
a, b, c (Å)	77.022, 87.519, 99.007	77.072, 87.094, 99.271
α, β, γ (°)	90.00, 90.00, 90.00	90.00, 90.00, 90.00
Resolution (Å)	43.13-1.42 (1.61-1.42)*	49.64-1.35 (1.49-1.35)*
Rmerge	0.199 (1.917)	0.124 (1.730)
I/σI	11.284 (1.838)	12.237 (1.776)
Completeness (%)	60.10 (9.63)	72.85 (14.91)
Spherical		
Completeness (%)	95.6 (75.68)	95.98 (73.43)
Elliptical		
Multiplicity	13.30 (11.49)	13.27 (11.05)
**Refinement**		
Resolution (Å)	43.13-1.42	49.64-1.35
No. of reflections	72951	100709
Rwork/Rfree	0.156 / 0.187	0.146 / 0.169
No. of atoms		
Protein	4853	4853
Ligand/ion	61	62
Water	579	643
B-factors		
Protein	16.13	16.96
Ligand/ion	23.00	23.29
Water	31.67	32.53
R.M.S deviations		
Bond lengths (Å)	0.010	0.017
Bond angles (°)	1.572	1.953
PDB ID	8AWH	8AVA

* Values in parentheses are for highest-resolution shell

Fig. S8.

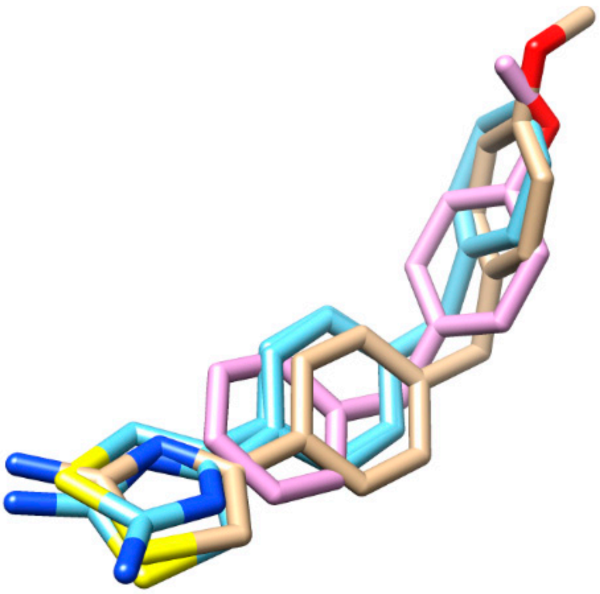

Fig. S9.

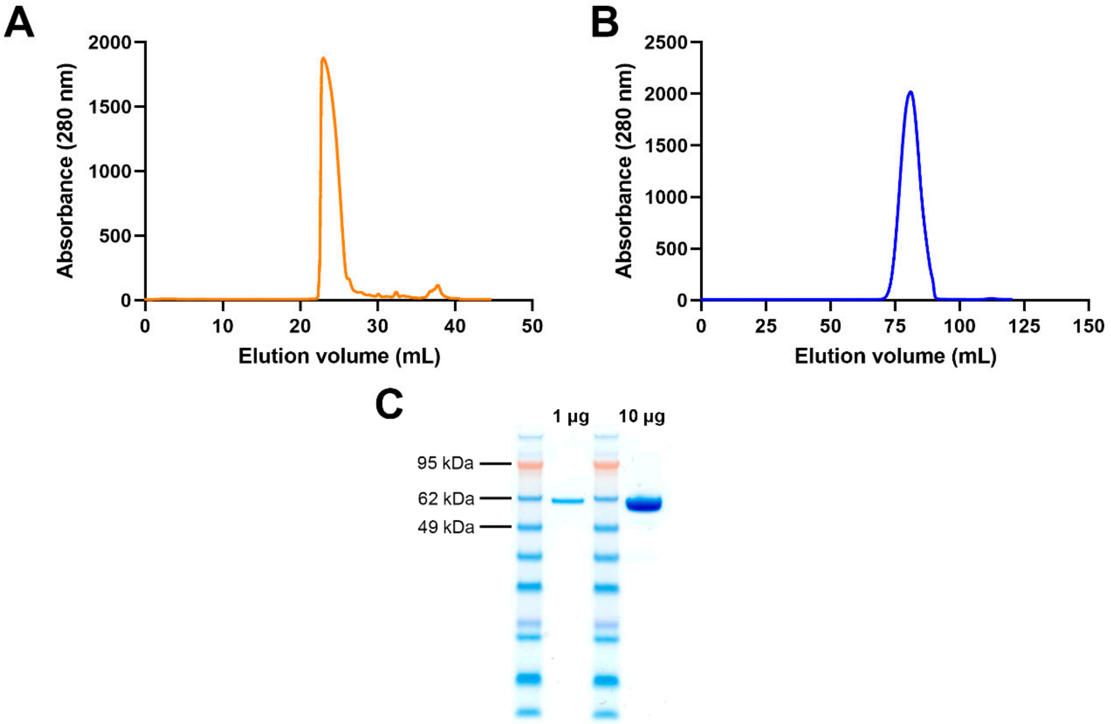
A
Absorbance (280 nm)
2000
1500
1000
500
0
0
10
20
30
40
50
Elution volume (mL)
B
Absorbance (280 nm)
2500
2000
1500
1000
500
0
0
25
50
75
100
125
150
Elution volume (mL)
C
1 µg
10 µg
95 kDa
62 kDa
49 kDa

Fig. S10.

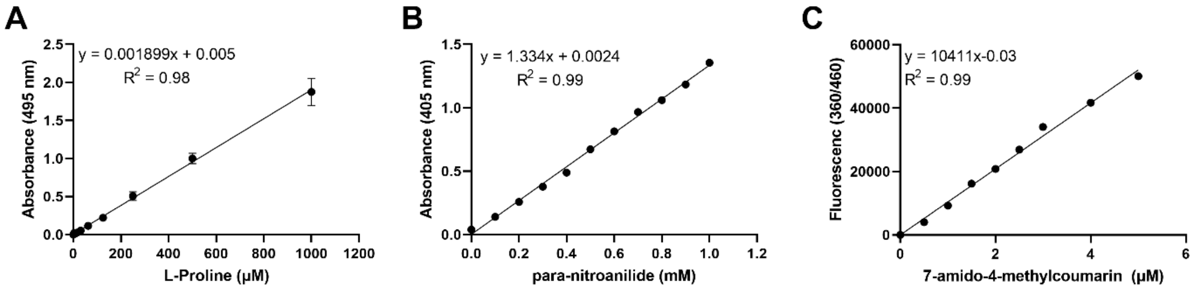
A
y = 0.001899x + 0.005
R2 = 0.98
Absorbance (495 nm)
L-Proline (μM)
B
y = 1.334x + 0.0024
R2 = 0.99
Absorbance (405 nm)
para-nitroanilide (mM)
C
y = 10411x-0.03
R2 = 0.99
Fluorescenc (360/460)
7-amido-4-methylcoumarin (μM)
